# Survival implications vs. complications: unraveling the impact of vitamin D adjunctive use in critically ill patients with COVID-19—A multicenter cohort study

**DOI:** 10.3389/fmed.2023.1237903

**Published:** 2023-08-24

**Authors:** Khalid Al Sulaiman, Ghazwa B. Korayem, Ohoud Aljuhani, Ali F. Altebainawi, Mohammad S. Shawaqfeh, Sumaiah J. Alarfaj, Reham A. Alharbi, Mawaddah M. Ageeli, Abdulrahman Alissa, Ramesh Vishwakarma, Alnada Ibrahim, Abeer A. Alenazi, Suliman Alghnam, Nadiyah Alshehri, Maqbulah M. Alshammari, Alaa Alhubaishi, Mohammed Aldhaeefi, Faisal F. Alamri, Yadullah Syed, Raymond Khan, Mai Alalawi, Khalaf A. Alanazi, Faisal S. Alresayes, Khalid J. Albarqi, Ghassan Al Ghamdi

**Affiliations:** ^1^Department of Pharmaceutical Care, King Abdulaziz Medical City, Riyadh, Saudi Arabia; ^2^College of Pharmacy, King Saud Bin Abdulaziz University for Health Sciences, Riyadh, Saudi Arabia; ^3^Population Health Section, King Abdullah International Medical Research Center, Riyadh, Saudi Arabia; ^4^Saudi Critical Care Pharmacy Research (SCAPE) Platform, Riyadh, Saudi Arabia; ^5^Department of Pharmacy Practice, College of Pharmacy, Princess Nourah bint Abdulrahman University, Riyadh, Saudi Arabia; ^6^Department of Pharmacy Practice, Faculty of Pharmacy, King Abdulaziz University, Jeddah, Saudi Arabia; ^7^Department of Clinical Pharmacy, College of Pharmacy, University of Hail, Hail, Saudi Arabia; ^8^Pharmaceutical Care Services, King Salman Specialist Hospital, Hail Health Cluster, Ministry of Health, Hail, Saudi Arabia; ^9^Department of Pharmacy, King Faisal Specialist Hospital and Research Center, Jeddah, Saudi Arabia; ^10^Department of Pharmacy, Prince Faisal Bin Khalid Cardiac Center PFKCC, Ministry of Health, Abha, Saudi Arabia; ^11^Pharmaceutical Care Services, King Abdullah Bin Abdulaziz University Hospital, Riyadh, Saudi Arabia; ^12^Department of Statistics, European Organization for Research and Treatment of Cancer (EORTC) Headquarters, Brussels, Belgium; ^13^Department of Pharmaceutical Care, Prince Sultan Military Medical City, Riyadh, Saudi Arabia; ^14^Department of Clinical and Administrative Pharmacy Sciences, College of Pharmacy, Howard University, Washington, DC, United States; ^15^Department of Basic Sciences, College of Science and Health Professions, King Saud Bin Abdulaziz University for Health Sciences, Jeddah, Saudi Arabia; ^16^King Abdullah International Medical Research Center, Jeddah, Saudi Arabia; ^17^Department of Pharmaceutical Care, King Abdulaziz Medical City, Jeddah, Saudi Arabia; ^18^Department of Respiratory, King Abdulaziz Medical City, Riyadh, Saudi Arabia; ^19^Department of Intensive Care, King Abdulaziz Medical City, Riyadh, Saudi Arabia; ^20^College of Medicine, King Saud Bin Abdulaziz University for Health Sciences, Riyadh, Saudi Arabia

**Keywords:** vitamin D, critically ill, COVID-19, SARS-CoV-2, intensive care units (ICUs), mortality, MV duration, bleeding

## Abstract

**Background:**

Despite insufficient evidence, vitamin D has been used as adjunctive therapy in critically ill patients with COVID-19. This study evaluates the effectiveness and safety of vitamin D as an adjunctive therapy in critically ill COVID-19 patients.

**Methods:**

A multicenter retrospective cohort study that included all adult COVID-19 patients admitted to the intensive care units (ICUs) between March 2020 and July 2021. Patients were categorized into two groups based on their vitamin D use throughout their ICU stay (control vs. vitamin D). The primary endpoint was in-hospital mortality. Secondary outcomes were the length of stay (LOS), mechanical ventilation (MV) duration, and ICU-acquired complications. Propensity score (PS) matching (1:1) was used based on the predefined criteria. Multivariable logistic, Cox proportional hazards, and negative binomial regression analyses were employed as appropriate.

**Results:**

A total of 1,435 patients were included in the study. Vitamin D was initiated in 177 patients (12.3%), whereas 1,258 patients did not receive it. A total of 288 patients were matched (1:1) using PS. The in-hospital mortality showed no difference between patients who received vitamin D and the control group (HR 1.22, 95% CI 0.87–1.71; *p* = 0.26). However, MV duration and ICU LOS were longer in the vitamin D group (beta coefficient 0.24 (95% CI 0.00–0.47), *p* = 0.05 and beta coefficient 0.16 (95% CI −0.01 to 0.33), *p* = 0.07, respectively). As an exploratory outcome, patients who received vitamin D were more likely to develop major bleeding than those who did not [OR 3.48 (95% CI 1.10, 10.94), *p* = 0.03].

**Conclusion:**

The use of vitamin D as adjunctive therapy in COVID-19 critically ill patients was not associated with survival benefits but was linked with longer MV duration, ICU LOS, and higher odds of major bleeding.

## Introduction

Since the emergence of COVID-19, the pandemic at the end of 2019 in Wuhan, China, several treatment modalities have been proposed as effective treatments for COVID-19 or adjunctive therapies for relieving patients' symptoms of COVID-19 (1). Adjunctive supplemental therapies, including vitamin C, vitamin D, thiamine, and zinc, have been used in critically ill patients with COVID-19 despite insufficient evidence (2).

Vitamin D is a fat-soluble vitamin that regulates the immune response and increases the synthesis of strong antimicrobial peptides (AMPs), therefore protecting the lungs against infection (3). The potent AMPs are found in neutrophils, monocytes, natural killer cells, and epithelial cells lining the respiratory tract and play a significant role in preventing lung infection. The expression of these potent AMPs is dramatically stimulated by the activated vitamin D, 1,25(OH)_2_D (4).

Vitamin D insufficiency is prevalent in intensive care unit (ICU) patients and is linked to higher disease severity, a longer ICU stay, and longer mechanical ventilation (MV) (5–7). In a subgroup analysis, ICU patients with severe vitamin D insufficiency who were treated with vitamin D had lower hospital mortality than patients with less severe vitamin D deficiency or placebo groups (8). Furthermore, a prospective observational study of asymptomatic and critically ill ICU patients discovered that vitamin D insufficiency greatly increases the risk of severe. Severe acute respiratory syndrome coronavirus 2 (SARS-CoV-2) illness (9). Another multicenter prospective cohort study of hospitalized patients with moderate-to-severe COVID-19 found that patients with severe vitamin D insufficiency stayed in the hospital longer than those with better vitamin D levels (10).

Vitamin D intake was linked to a lower incidence of respiratory-related infections, including non-influenza respiratory viral infections, and rhinovirus (11–13). However, randomized controlled trials (RCTs) found that high-dosage vitamin D supplementation in non-COVID-19 ICU patients was not associated with lower mortality or better patient outcomes when compared with placebo (8, 14).

A meta-analysis and a systematic review found that vitamin D may have a role in lowering the severity of symptoms in COVID-19 hospitalized patients (15, 16). While vitamin D has been shown to have a potential effect as an additional therapy for COVID-19, an observational study found no mortality benefit when vitamin D supplementation was administered to ICU patients with vitamin D insufficiency (17).

Given the inadequate evidence and contradictory findings, the efficacy of vitamin D in COVID-19 ICU patients remains debatable. Therefore, this study aims to assess the role of vitamin D supplementation as an adjunctive therapy in COVID-19 ICU patients.

## Methods

This study is part of the Saudi Critical Care Pharmacy Research (SCAPE) platform, which conducted several studies that evaluated the safety and effectiveness of multiple therapies in critically ill patients (18). The design of this multicenter cohort study is similar to previously published studies (19–25). The details of the study design are available in Supplementary material.

### Study design

This is a multicenter, retrospective cohort study of adult patients with COVID-19 who were admitted to the ICUs between 1 March 2020 and 31 July 2021. COVID-19 was identified using reverse transcriptase-polymerase chain reaction (RT-PCR) nasopharyngeal or throat swabs. Vitamin D use during the ICU stay was prescribed empirically, not based on levels, as there were no predefined criteria for vitamin D initiation. The included patients were classified based on their vitamin D administration throughout their ICU stay. Patients who received vitamin D during their ICU admission were the active group, while patients who did not receive it were defined as controls. All patients were followed until they were discharged from the hospital or died during the in-hospital stay, whichever came first. The King Abdullah International Medical Research Center (KAIMRC) approved the study in February 2022 (Ref. No. NRC22R/045/01).

### Study participants

**Figure 1:**
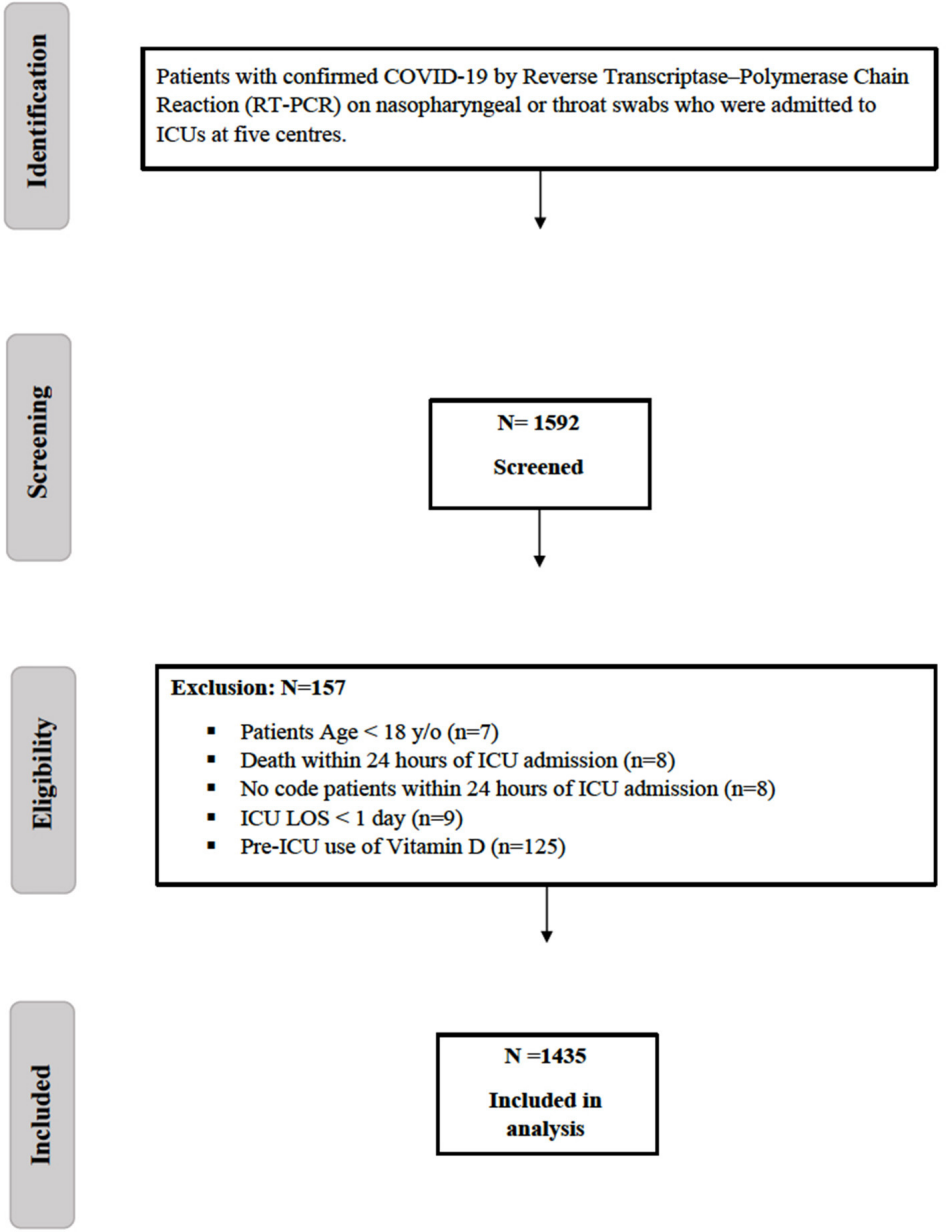
Flow diagram showing patients recruited with COVID-19. COVID-19, Coronavirus disease; ICU, intensive care unit; LOS, length of stay.

All adult patients (age ≥18 years) admitted to the ICUs with confirmed COVID-19 were screened for eligibility. Patients were excluded if they received vitamin D before ICU admission, had an ICU length of stay (LOS) of ≤ 1 day, died within the first 24 h of ICU admission, or were labeled as “Do-Not-Resuscitate” (Figure 1).

### Study setting

The study was conducted at five medical facilities and medical cities in Saudi Arabia; details of participating hospitals and the leading centers can be found in Supplementary File 1.

### Data collection

Variables and data were collected using the Research Electronic Data Capture (REDCap^®^) platform that included demographic data, comorbidities, laboratory data, vital signs, and baseline severity. Details of the collected data can be found in Supplementary File 1.

### Outcomes

The primary endpoint was in-hospital mortality. The secondary endpoints were 30-day mortality, hospital LOS, ICU LOS, and MV duration. The ICU-acquired complications were considered exploratory outcomes, such as respiratory failure requiring MV, thrombosis, bleeding, new-onset atrial fibrillation, AKI, liver injury, hospital/ventilator-acquired pneumonia, and secondary fungal infection (Additional File 1).

### Sample size calculation

The sample size was calculated using MedCalc Software Version 20.116. A two-group chi-squared test with a 0.05 two-sided significance level will have 80% power to detect the anticipated difference between the prevalence of mortality, which was 0.24 (19). The sample size in each group is 246. A total sample size of 492 was considered to assess the study's primary endpoint.

### Statistical analysis

We presented continuous variables utilizing means and standard deviations (SDs) or medians with lower and upper quartiles (Q1–Q3) as appropriate, while categorical variables as counts and percentages. The normality assumptions were evaluated using the Shapiro–Wilk test. Propensity score matching procedure (Proc PS match) (SAS, Cary, NC) was used to match patients (1:1 ratio) who received Vitamin D therapy (active group) to patients who did not (control group) based on patient's APACHE II scores, AKI, proning status and the early use of dexamethasone within 24 hours of ICU admission.

Multivariable Cox proportional hazards regression analyses, multivariable regression analyses, and negative binomial regression were used for the outcomes considered in this study. Regression analysis was conducted by considering the PS score as one of the covariates in the model. The odds ratios (ORs), hazard ratios (HRs), or estimates with 95% confidence intervals (CIs) were reported as appropriate. We considered a *P*-value of < 0.05 statistically significant and used SAS version 9.4 for all statistical analyses (Supplementary File 1).

## Results

A total of 1,592 critically ill patients with COVID-19 were screened, and 1,435 were eligible for inclusion (Figure 1). Vitamin D supplement was newly initiated in the ICU for 177 patients, whereas 1,258 patients did not receive vitamin D as an adjunctive therapy during their ICU stay. We matched 288 patients using a propensity score (1:1) according to the selected criteria. The median time for vitamin D initiation was 1 (0.00, 5.00) days from ICU admission. The most common type of vitamin D was cholecalciferol, accounting for 97.9% of the prescribed vitamin D supplements, of which 8.3% was in the form of combination therapy of calcium carbonate/cholecalciferol, followed by 2.1% as alfacalcidol. The median dose of cholecalciferol was 5,000 (2800, 7142) U/day with a median duration of 14 (8.0, 18.0) days. Among the patients included in the study, only 10.8% underwent vitamin D level assessments while in the ICU. Among those who were assessed, 41.9% had insufficient vitamin D levels (25–50 nmol/L), followed by 19.4% with sufficient vitamin D levels and 9.6% with a deficiency of vitamin D (<25 nmol/L).

### Demographic and clinical characteristics

The majority of the patients in both arms were men (63.3%), and the average age was 62 ± 14.86 years. The most prevalent comorbidities were diabetes mellitus (59.6%), followed by hypertension (57.7%) and dyslipidemia (21.3%) (Table 1). Moreover, there was a significant difference in the baseline characteristics between the two groups before PS. Furthermore, the average weight, BMI, early use of dexamethasone, and use of pharmacological DVT prophylaxis and nephrotoxic medications during the ICU stay were higher in the vitamin D group than in the control group. On the flip side, nutritional status based on the Nutrition Risk in the Critically Ill (NUTRIC) Score, AKI within 24 h of ICU admission, and International Normalized Ratio (INR), albumin, and ferritin levels as a baseline were higher in the control group. However, after PS matching based on the selected criteria, most baseline characteristics and comorbidities were balanced between the two groups except for alanine transaminase (ALT) at admission and the use of nephrotoxic drugs/materials during the ICU stay, which were significantly higher in the active group. Severity scores (APACHE II, SOFA, and multiple organ dysfunction scores) at admission before and after PS matching were not statistically different between the two groups (Table 1). More baseline characteristics are reported in Additional File 2.

**Table 1:** Summary of demographics and baseline characteristics.

	**Before propensity score (PS)**				**After propensity score (PS)**			
	**Overall (*****N** =* **1,435)**	**Control (*****N** =* **1,258)**	**Vitamin D (*****N** =* **177)**	***P*** **-value**	**Overall (*****N** =* **288)**	**Control (*****N** =* **144)**	**Vitamin D (*****N** =* **144)**	***P*** **-value**
Age (years), mean (SD)	62.0 (14.86)	62.0 (15.01)	61.6 (13.79)	0.6678^∧^	62.1 (15.40)	63.3 (16.58)	60.9 (14.06)	0.1818^*^
Sex—male, *n* (%)	880 (63.3)	770 (63.2)	110 (63.6)	0.9257^∧∧^	187 (65.4)	92 (64.3)	95 (66.4)	0.7092^∧∧^
Weight (kg), mean (SD)	81.2 (18.73)	80.7 (18.62)	84.6 (19.25)	0.0146^∧^	82.3 (19.95)	80.7 (20.62)	84.0 (19.19)	0.1518^∧^
Body mass index (BMI), mean (SD)	30.9 (8.48)	30.7 (8.52)	32.2 (8.06)	<0.01^∧^	31.2 (7.68)	30.7 (7.38)	31.8 (7.96)	0.3392^∧^
APACHE II score, median (Q1, Q3)	14.0 (9.00, 21.00)	14.0 (9.00, 22.00)	12.0 (9.00, 19.00)	0.0863^∧^	12.0 (9.00, 20.00)	12.0 (10.00, 20.00)	12.0 (9.00, 20.00)	0.8736^∧^
SOFA score, median (Q1, Q3)	5.0 (3.00, 7.00)	5.0 (3.00, 7.00)	5.0 (3.00, 7.00)	0.7950^∧^	4.0 (3.00, 7.00)	4.0 (2.00, 6.00)	5.0 (3.00, 7.00)	0.0987^∧^
NUTRIC score, median (Q1, Q3)	3.0 (2.00, 5.00)	3.0 (2.00, 5.00)	3.0 (2.00, 4.00)	0.0116^∧^	3.0 (2.00, 5.00)	3.0 (2.00, 5.00)	3.0 (2.00, 4.00)	0.3235^∧^
Multiple organ dysfunction score at admission, median (Q1, Q3)	5.0 (4.00, 7.00)	5.0 (4.00, 7.00)	5.0 (4.00, 7.00)	0.6080^∧^	5.0 (4.00, 7.00)	5.0 (4.00, 7.00)	5.0 (4.00, 7.00)	0.4563^∧^
Total 25-OH vitamin D level during stay (nmol/L), median (Q1, Q3)	39.9 (30.00, 64.90)	41.2 (30.20, 63.00)	39.6 (30.00, 65.40)	0.8780^∧^	39.9 (31.00, 57.35)	48.9 (38.23, 90.79)	39.6 (30.78, 57.35)	0.3610^∧^
Adjusted calcium (mmol/L), median (Q1, Q3)	2.2 (2.08, 2.26)	2.2 (2.07, 2.26)	2.2 (2.09, 2.29)	0.5308^∧^	2.2 (2.09, 2.28)	2.2 (2.08, 2.25)	2.2 (2.10, 2.30)	0.2723^∧^
Serum creatinine (mmol/L) at admission, median (Q1, Q3)	88.0 (68.00, 132.00)	88.0 (68.00, 134.00)	82.0 (68.00, 121.00)	0.1544^∧^	83.5 (68.00, 115.00)	84.6 (67.00, 109.00)	81.5 (68.00, 121.00)	0.8747^∧^
Blood urea nitrogen (BUN) at admission (mmol/L), median (Q1, Q3)	7.0 (4.80, 11.80)	7.0 (4.86, 11.90)	6.9 (4.50, 10.80)	0.2316^∧^	7.0 (4.80, 10.10)	7.0 (4.84, 9.90)	6.9 (4.70, 10.40)	0.9854^∧^
Acute kidney injury (AKI) within 24 hours of ICU admission, *n* (%)	399 (28.8)	361 (29.8)	38 (21.8)	0.0309^∧∧^	55 (19.2)	27 (18.9)	28 (19.6)	0.8807^∧∧^
**Comorbidity**, ***n*** **(%)**								
Atrial fibrillation (A Fib)	58 (4.1)	50 (4.1)	8 (4.6)	0.7544^∧∧^	12 (4.2)	7 (4.9)	5 (3.5)	0.5553^∧∧^
Heart failure	116 (8.3)	98 (8.0)	18 (10.3)	0.2987^∧∧^	19 (6.6)	5 (3.5)	14 (9.8)	0.0326^∧∧^
Hypertension	810 (57.7)	713 (58.0)	97 (55.4)	0.5171^∧∧^	160 (55.9)	85 (59.4)	75 (52.4)	0.2336^∧∧^
Diabetes mellitus	837 (59.6)	739 (60.1)	98 (56.0)	0.2975^∧∧^	160 (55.9)	86 (60.1)	74 (51.7)	0.1529^∧∧^
Dyslipidemia	299 (21.3)	254 (20.7)	45 (25.7)	0.1271^∧∧^	65 (22.7)	32 (22.4)	33 (23.1)	0.8878^∧∧^
Ischemic heart disease (IHD)	135 (9.6)	120 (9.8)	15 (8.6)	0.6166^∧∧^	24 (8.4)	16 (11.2)	8 (5.6)	0.0880^∧∧^
Chronic kidney disease (CKD)	168 (12.0)	151 (12.3)	17 (9.7)	0.3267^∧∧^	27 (9.4)	13 (9.1)	14 (9.8)	0.8397^∧∧^
Cancer	64 (4.6)	57 (4.6)	7 (4.0)	0.7050^∧∧^	14 (4.9)	10 (7.0)	4 (2.8)	0.1001^∧∧^
Deep vein thrombosis (DVT)	14 (1.0)	13 (1.1)	1 (0.6)	0.5446^**^	2 (0.7)	1 (0.7)	1 (0.7)	>0.9999^**^
Pulmonary embolism (PE)	12 (0.9)	11 (0.9)	1 (0.6)	0.6635^**^	2 (0.7)	1 (0.7)	1 (0.7)	>0.9999^**^
Liver disease (any type)	31 (2.2)	25 (2.0)	6 (3.4)	0.2402^**^	4 (1.4)	0 (0.0)	4 (2.8)	0.0440^**^
Stroke	87 (6.2)	80 (6.5)	7 (4.0)	0.1977^∧∧^	16 (5.6)	11 (7.7)	5 (3.5)	0.1226^∧∧^

^*^T-Test / ^∧^Wilcoxon rank sum test is used to calculate the P-value. ^∧∧^Chi-square/ ^**^Fisher's exact test is used to calculate the P-value.

### 30-day and in-hospital mortality

There were 66 patients (56.4%) who died within 30 days among the vitamin D group, compared with 53 patients (41.4%) in the control group after propensity score matching, which was statistically significant (*P* = 0.02). However, in the multivariable Cox proportional hazards regression analysis, the 30-day mortality was not significantly different (HR 1.06, 95% CI 0.73, 1.52: *P* = 0.76). Similarly, the in-hospital mortality was not statistically different between the two groups (HR 1.22, 95% CI 0.87, 1.71; *p* = 0.26; Table 2).

**Table 2:** The outcomes of critically ill patients with COVID-19 after propensity score matching.

**Outcomes**	**Number of outcomes/total number of patients**			**Hazard ratio (HR) (95%CI)**	***p*-value^$^**
	**Control**	**Vitamin D**	***p*** **-value** ^∧∧^		
30-day mortality, *n* (%)^Δ^	53 (41.4)	66 (56.4)	0.02^∧∧^	1.06 (0.73, 1.52)	0.76
In-hospital mortality, *n* (%)^Δ^	62 (47.7)	72 (58.1)	0.09^∧∧^	1.22 (0.87, 1.71)	0.26
			***p*** **-Value** ^∧^	**beta coefficient (Estimates) (95%CI)**	***p*** **-Value** ^$*^
MV duration (days), median (Q1, Q3)^Δ^	9.0 (3.00, 16.00)	12.0 (6.00, 20.00)	<0.01^∧^	0.24 (0.00, 0.47)	0.05
ICU length of stay (days), median (Q1, Q3)^Δ^	12.0 (5.00, 19.00)	15.0 (9.00, 24.00)	0.02^∧^	0.16 (−0.01, 0.33)	0.07
Hospital length of stay (days), median (Q1, Q3)^Δ^	18.0 (10.00, 29.00)	20.0 (13.00, 27.00)	0.42^∧^	0.00 (−0.17, 0.17)	0.99

^Δ^Denominator of the percentage is the total number of patients. ^∧^The Wilcoxon rank sum test is used to calculate the P-value. ^∧∧^The chi-squared test is used to calculate the P-value. ^$^Cox proportional hazards regression analysis used to calculate HR and p-value. ^$*^Generalized linear model is used to calculate estimates and p-value.

### MV duration and length of stay

Patients who received vitamin D have a statistically significant longer MV duration than patients who did not [median (Q1, Q3) 12 (6, 20) vs. 9 (3, 16) days; *P* = <0.01]. The regression analysis was also significantly longer [beta coefficient 0.24 (95% CI 0.00, 0.47), *P* = 0.05]. Similarly, the median ICU LOS was significantly longer in crude analysis [median (Q1, Q3) 15.0 (9.0, 24.0) vs. 12.0 (5.0, 19.0) days; *P* = 0.02] than the control group; however, it did not reach the statistical significance at regression analysis [beta coefficient 0.16 (95% CI −0.01 to 0.33), *P* = 0.07]. Moreover, the hospital LOS [median (Q1, Q3) 20.0 (13.00, 27.00) vs. 18.0 (10.00, 29.00) days, *P* = 0.42] was not statistically different between the patients who received vitamin D as the adjunctive therapy and the control group [beta coefficient 0.00 (95% CI −0.17 to 0.17), *P* = 0.99] (Table 2).

### Complications during the ICU stay

As per exploratory outcomes, thirteen patients developed major bleeding in the vitamin D group compared to four patients in the control group (9.6 vs. 2.9%; *p* = 0.02). The median time for major bleeding from vitamin D initiation was 13.5 (2.5, 30.5) days. The logistic regression analysis showed a statistically significant difference in which those who used vitamin D were over three times more likely to develop major bleeding than those who did not [OR 3.48 (95% CI 1.10, 10.94), *p* = 0.03] (Table 2) despite the similar use of pharmacological DVT prophylaxis, the intensity of DVT prophylaxis dosing, and aspirin during the ICU stay (Additional File 2). In addition, minor bleeding during the ICU stay was higher in the vitamin D group; however, it was not statistically significant [OR 1.33 (95% CI 0.52, 3.36), *p* = 0.55].

Patients who received vitamin D as an adjunctive therapy during the ICU stay were more likely to have hospital/ventilator-acquired pneumonia than the control group [OR 1.71 (95% CI 1.05, 2.77), *p* = 0.03]. However, developing secondary fungal infections did not differ between the two groups [OR 1.04 (95% CI 0.53, 2.02), *p* = 0.89]. Other outcomes such as new-onset atrial fibrillation, thrombosis, AKI, and liver injury during the ICU stay were not statistically different between both groups in the crude analysis and the logistic regression analysis (Table 3).

**Table 3:** The ICU complications during stay.

**Outcomes**	**Number of outcomes/total number of patients**		***P*-value**	**Odds ratio (OR) (95%CI)**	*P*-value^$*^
	**Control**	**Vitamin D**			
New-onset A fib., *n* (%)^Δ^	14 (9.8)	9 (6.3)	0.28^∧∧^	0.62 (0.258, 1.479)	0.28
Acute kidney injury, *n* (%)^Δ^	55 (38.5)	60 (42.0)	0.55^∧∧^	1.20 (0.739, 1.964)	0.46
Liver injury, n (%)^Δ^	19 (13.3)	18 (12.6)	0.86^∧∧^	0.94 (0.471, 1.875)	0.86
Thrombosis, *n* (%)^Δ^	12 (8.4)	18 (12.6)	0.25^∧∧^	1.57 (0.728, 3.397)	0.24
Major bleeding, *n* (%)^Δ^	4 (2.9)	13 (9.6)	0.02^∧∧^	3.48 (1.104, 10.948)	0.03
Minor bleeding, *n* (%)^Δ^	9 (6.6)	10 (7.5)	0.77^∧∧^	1.33 (0.526, 3.369)	0.55
Hospital/ventilator-acquired pneumonia, *n* (%)^Δ^	48 (33.6)	65 (45.5)	0.04^∧∧^	1.71 (1.056, 2.771)	0.03
Secondary fungal infection, *n* (%)^Δ^	21 (15.2)	21 (15.3)	0.98^∧∧^	1.04 (0.538, 2.024)	0.89

^Δ^Denominator of the percentage is the total number of patients. ^∧∧^The chi-squared test is used to calculate the P. ^$*^Logistic regression is used to calculate the OR and p-value.

## Discussion

This study was conducted to evaluate the role of vitamin D supplementation as an adjunctive therapy in patients with critically ill COVID-19 (CI-COVID-19) and found that vitamin D was not associated with reduced mortality or hospital and ICU LOS. Moreover, the adjunctive vitamin D group had higher odds of major bleeding events and hospital/ventilator-acquired pneumonia with a prolonged MV duration.

Vitamin D has been suggested as a facilitator of immune competence in COVID-19 infection due to its role in promoting innate and adaptive immune systems and its antiviral activity. Additionally, vitamin D helps regulate the cytokine storm associated with major pathophysiologic aspects of COVID-19 infection.

The presumed benefits of vitamin D adjunctive therapies have attracted clinicians after several studies addressed the association between low vitamin D levels and increased mortality in hospitalized COVID-19 patients (26, 27). A systematic review and meta-analysis (SRMA) by Teshone et al. (28), which included a pooled analysis of 91,120 patients, found that being vitamin D deficient increased the risk of contracting COVID-19 infection by 80%, compared to vitamin D sufficiency (28). In addition, another SRMA by Wan et al. (27) that included 2,756 patients reported that vitamin D deficiency was associated with increased mortality, hospital admission, and a longer hospital stay than patients with no vitamin D deficiency (27).

Our cohort came from several centers in Saudi Arabia, in a population with known low levels of vitamin D (up to 60%), according to a recent meta-analysis (29). The question still remains whether the absence of survival benefits in our group can be attributed to a pre-existing deficiency of vitamin D in our population. Therefore, providing vitamin D as an additional therapy for a limited duration during ICU admission may not result in the same preventive effects observed in earlier studies. Lacking clear benefits of the adjunctive therapy in our cohort might stimulate further research in critically ill patients with normal vitamin D levels to measure the isolated impact of adjunctive vitamin D on COVID-19.

Studies investigating the role of vitamin D supplementation on the risk of mortality and severity of the disease were underpowered to detect a difference and have yielded mixed results (30–36). In 2021, a study by Murai et al. (32) evaluated the effect of vitamin D supplementation on moderately to severely ill COVID-19 patients and found that it did not decrease LOS, mortality, ICU admission, or the need for MV (32). Another study by Castillo et al. (33), conducted in 2020, found that vitamin D supplementation in hospitalized patients decreased the risk of ICU admission and mortality (33). An SRMA study by Rawat et al. included data from 467 patients and found that vitamin D supplementation did not decrease mortality, ICU admission, or the need for MV (34).

A recent SRMA by Pal et al. (36) reviewed 13 studies evaluating the effect of vitamin D supplementation on clinical outcomes, including ICU admission and mortality in hospitalized COVID-19 patients, and the pooled data of 2,933 patients indicated an improvement in clinical outcomes (36). However, most of the included studies did not report the severity of COVID-19 infection and excluded ICU-admitted patients from their products.

Studies investigating the role of vitamin D supplementation in patients with CI-COVID-19 are limited. Our results are consistent with a prospective observational cohort study by Guven et al., which investigated the effect of administering a high parenteral dose of vitamin D3 (300,000 IU intramuscularly) to COVID-19 patients admitted to ICUs and found no mortality benefit (17). They attributed the lack of benefit to their patient population's advanced age, malnutrition, and comorbidities. Additionally, they interpreted that vitamin D did not have enough time to show its effect due to rapid progression to respiratory failure and death. Compared to the previous cohort, the mean age of patients in the current study was 62 years, with normal nutrition scores. Still, our results are limited by not having the pre-admission vitamin D level, which may help accurately reflect nutritional status, especially in a population with known low levels of vitamin D (29). Additionally, the lack of benefit in our study could be attributed to the recent initiation of vitamin D in the ICU, which could limit the benefits observed with its prolonged use.

The findings of this study suggest a negative association between vitamin D administration and one of our exploratory outcomes (i.e., bleeding events) in critically ill patients with COVID-19. The effect of COVID-19 on hemostasis and the immune system has been proposed as a potential mechanism for COVID-19-related thrombosis and bleeding (37–39).

Apart from its immunomodulatory effects, vitamin D is recognized for its antithrombotic properties. It enhances the expression of natural anticoagulants like thrombomodulin and deactivates tissue factor, thereby reducing the hypercoagulable state. This mechanism may interfere with one of the proposed pathways in COVID-19 that contributes to thrombosis (40, 41). Generally, patients with COVID-19 are at risk of coagulation disorders that involve thromboembolic events or bleeding (42). A multifactorial etiology has been hypothesized for bleeding in COVID-19 patients (43). Recent evidence suggests that vitamin D exerts anticoagulant effects, which might have a direct or indirect effect on increasing bleeding risk (44). Several clinical trials have investigated vitamin D as a potential therapeutic option for prophylaxis and treatment of thromboembolic events, which highlights the potential effect of vitamin D as an anticoagulant (45, 46). In addition, vitamin D metabolites were found to have antithrombotic effects and were postulated to act as anticoagulants in the body (46–49). Thus, the administration of vitamin D during the COVID-19 infection might explain the increased bleeding events. However, since the pre-admission levels of vitamin D were unavailable for most patients and given the lack of standardization of vitamin D administration in this study, it is difficult to further explain the effect of vitamin D supplementation on bleeding.

Additionally, the results of this study indicate that vitamin D use was associated with an increased MV duration and increased events of ventilator-acquired pneumonia. In contrast, other studies reported that patients with low levels of 25(OH)D were more likely to need invasive MV (50), and vitamin D supplementation in critically ill patients showed inconsistent results on the duration of MV (35, 36). An SRMA study by Hariyanto et al. evaluated the effect of vitamin D supplementation on COVID-19 patients and found a decreased need for MV (51). However, only a few studies assessed the effect of vitamin D administration on the duration of MV in critically ill patients. In 2019, a study showed a trend to decrease the duration of MV, which was not statistically significant (52), while the other study, published in 2016, showed no benefit in reducing MV duration (53). This increase in MV duration observed in our study could be attributed to differences in baseline characteristics, including more heart failure patients in the vitamin D group. Additionally, the increased bleeding and pneumonia in the vitamin D group could have led to an increased duration of MV.

The findings of this study should be interpreted while considering some limitations. These include the retrospective observational single-nation design of this study, the small sample size, and the risk of type 1 error. Furthermore, vitamin D initiation in our centers was primarily based on clinical judgment rather than a standardized protocol, and vitamin D levels were only measured for a limited number of patients (only 10.8%), leading to the possibility of treating physicians' bias. Moreover, there was variation in the dose, duration, and forms of vitamin D supplementation, all of which may limit the study interpretation of the expected benefits in COVID-19 patients. The study has certain limitations due to the limited follow-up period for patients following vitamin D administration, which hinders the exploration of the extended effects of vitamin D beyond the ICU setting. Nonetheless, the study possesses various strengths. Firstly, its multi-center nature enhances the generalizability of the findings. Additionally, the study conducted a comprehensive evaluation of potential confounding factors and employed propensity score matching to minimize and control for these confounding effects, although it may not account for residual confounders. Therefore, prospective randomized trials are required to delve deeper into the effects of vitamin D on critically ill patients with COVID-19.

## Conclusion

The use of vitamin D in critically ill patients with COVID-19 was not associated with survival benefits but was linked with longer MV duration, ICU LOS, and higher odds of major bleeding. Further randomized clinical studies are required to evaluate and confirm these findings.

## Data availability statement

The raw data supporting the conclusions of this article will be made available by the authors, without undue reservation.

## Ethics statement

The study was approved in January 2022 by the King Abdullah International Medical Research Center (KAIMRC)-Institutional Review Board (IRB), Riyadh, Saudi Arabia (Ref. # NRC22R/045/01). All methods were performed in accordance with relevant guidelines and regulations. Participants' confidentiality was strictly observed throughout the study by using anonymous, unique serial numbers for each subject and restricting data only to the investigators. The KAIMRC-IRB committee waived informed consent due to its retrospective nature.

## Author contributions

KA and OA equally contributed to the conception and design of the research. GK and RV contributed to the acquisition and analysis of the data. AFA, MS, SA, and RA contributed to the interpretation of the data. MAg, AAli, AI, AAA, SA, NA, MAls, AAlh, and MAld drafted the manuscript. FFA, YS, RK, MAla, KAA, FSA, KJA, and KA critically revised the manuscript. All authors agree to be fully accountable for ensuring the integrity and accuracy of the study and have read and approved the final manuscript.
