# Supplementary material for: Survival implications vs. complications: unraveling the impact of vitamin D adjunctive use in critically ill patients with COVID-19—A multicenter cohort study
Source: Front Med (Lausanne). 2023 Aug 24;10:1237903. doi: 10.3389/fmed.2023.1237903 (PMC10484515; doi:10.3389/fmed.2023.1237903)
Supplement: Supplementary file 1 [file Presentation_1.pdf]

Supplementary file 1: Detailed methods

Study setting:

The study took place at five hospitals in Saudi Arabia: King Abdulaziz Medical City (Riyadh), King Abdulaziz Medical City (Jeddah), King Abdulaziz University Hospital (Jeddah), King Abdullah bin Abdulaziz University Hospital (KAAUH) (Riyadh), and King Salman Specialist Hospital (Hail). We included both tertiary and secondary centers. These centers were selected based on geographic distribution, availability of electronic records, and the center's willingness to participate in the national project. The primary site for this multicenter retrospective study was King Abdulaziz Medical City (Riyadh) which is considered a tertiary care center.

Data collection

Each patient's data was collected and handled using Research Electronic Data Capture (REDCap®) software hosted by King Abdullah International Medical Research Center (KAIMRC). We collected demographic information, comorbidities, vital signs and laboratory tests, severity scores (APACHE II, SOFA, NUTRIC, and multiple organ dysfunction scores), Glasgow Coma Score (GCS), acute kidney injury (AKI), use of prone positioning, and receipt of MV and MV parameters (e.g., PaO_2/FiO_2 ratio, FiO_2 requirement) within 24 hours of ICU admission. Furthermore, within 24 hours of ICU admission, a renal profile, liver function tests (LFTs), coagulation profile (i.e., INR, aPTT, fibrinogen, D-dimer), and other markers (Ferritin, procalcitonin, and creatine phosphokinase (CPK)) were collected. Vitamin D level and use (type, dose, and duration) were recorded for all patients at any point during their ICU stay. In addition,

Tocilizumab, corticosteroids, and pharmacological VTE prophylaxis use were recorded for the eligible patients.

Statistical analysis

We summarized numerical variables (continuous variables) utilizing means and standard deviations (SD), or medians and lower quartiles and upper quartiles (Q1-Q3), and categorical variables as counts and percentages. The normality assumptions were evaluated using the Shapiro-Wilk test, as well as through graphical techniques, for example, histograms and Q plots for all continuous variables . The validity of the statistical model assessed using the Hosmer-Lemeshow goodness-of-fit test.

The two study groups' baseline characteristics were compared. We compared normally distributed numerical variables with the t-test, and other continuous variables with the Mann-Whitney U test and categorical variables using the chi-square / Fisher exact test .

Multivariable Cox proportional hazards regression analyses used to find out the relationship between treatments and 30-day mortality. Before fitting the cox model, the proportionality assumption was assessed. Plotting a log(-log) plot performed to assess the assumption visually and also by testing the correlation of scaled Schoenfeld residuals with rank-ordered time.

Propensity score matching procedure (Proc PS match) (SAS, Cary, NC) was used to match patients who received Vitamin D therapy (active group) to patients who did not (control group) based on patient’s APACHE II scores, AKI, proning status and the early use of dexamethasone within 24 hours of ICU admission. The APACHE II scores and AKI were selected as covariates to reflect patients’ severity of illness. Proning status and dexamethasone use to determine if treatment

strategies had any effect on clinical outcomes. Greedy nearest neighbor matching method was used in which one patient who received vitamin D (active) matched with one patient who did not (control), which eventually produced the smallest within-pair difference among all available pairs with treated patients. Patients were matched only if the difference in the logits of the propensity scores for pairs of patients from the two groups was less than or equal to 0.5 times the pooled estimate of the standard deviation.

Multivariable regression analysis and negative binomial regression were used for the other outcomes considered in this study. Regression analysis was done by considering the PS score as one of the covariates in the model. The odds ratios (OR), hazard ratio (HR), or estimates with the 95% confidence intervals (CI) were reported as appropriate. No imputation was made for missing data as the cohort of patients in our study was not derived from random selection. We considered a *P*-value of < 0.05 statistically significant and used SAS version 9.4 for all statistical analyses.
