# Supplementary material for: Survival implications vs. complications: unraveling the impact of vitamin D adjunctive use in critically ill patients with COVID-19—A multicenter cohort study
Source: Front Med (Lausanne). 2023 Aug 24;10:1237903. doi: 10.3389/fmed.2023.1237903 (PMC10484515; doi:10.3389/fmed.2023.1237903)
Supplement: Supplementary file 2 [file Data_Sheet_1.docx]

**Additional file 1: Outcome definition(s)**

- In-hospital mortality was defined as percentage of patients with COVID-19 who died in the hospital
- The 30-day mortality was defined as a death occurring for any cause within 30 days of the admission date during hospital stay; patients who were discharged from the hospital alive were presumed to be survived.
- Acute kidney injury (AKI) was defined as a sudden decrease of renal function within 48 hours, defined by an increase in absolute SCr of at least 26.5 μmol/L (0.3 mg/dL) or by a percentage increase in SCr ≥ 50% (1.5× baseline value) during ICU stay (20).
- Acute liver injury was defined as alanine aminotransferase (ALT) exceeding three times the upper limit of normal or double in patients with elevated baseline ALT during the ICU stay.
- Secondary fungal infection was identified through the blood, urine, wound, drainage, cerebrospinal fluid, and/or respiratory cultures. Cultures were excluded if the laboratory reported them as a "contaminant sample."The fungal growth was considered significant if the growth was ≥ 100,000 colony forming units (CFUs)/ml in sputum or endotracheal aspiration, ≥10,000 CFUs of single organism/ml in bronchoalveolar lavage or ≥ 1000 CFUs of single organism/ml in protected specimen brushes. Additionally, urinary cultures were considered significant if showing a growth ≥100,000 CFUs/ml of no more than two species of microorganisms.
- Respiratory failure was defined as either low arterial carbon dioxide tension (PaCO_2_) or hypoxemic respiratory failure (PaO_2_ < 60 mm Hg with a normal or hypercapnic respiratory failure (PaCO_2_ > 50 mm Hg) that requires mechanical ventilation.
- Major bleeding defined according to the International Society on Thrombosis and Hemostasis (ISTH) definition. Any patient not fulfilling the criteria of major or clinically significant bleeding was identified as having a minor bleed.
