# Supplementary material for: Survival implications vs. complications: unraveling the impact of vitamin D adjunctive use in critically ill patients with COVID-19—A multicenter cohort study
Source: Front Med (Lausanne). 2023 Aug 24;10:1237903. doi: 10.3389/fmed.2023.1237903 (PMC10484515; doi:10.3389/fmed.2023.1237903)
Supplement: Supplementary file 3 [file Data_Sheet_2.docx]

**Additional file 2: Summary of additional Baseline characteristics**

|  | **Before propensity score (PS)** | | | | **After propensity score (PS)** | | | |
| --- | --- | --- | --- | --- | --- | --- | --- | --- |
|  | **Overall (N=1435)** | **Control (N=1258)** | **Vitamin D (N=177)** | **P-value** | **Overall (N=288)** | **Control (N=144)** | **Vitamin D (N=144)** | **P-value** |
| **Early use of Dexamethasone within 24 hours, n (%)** | 867 ( 61.8 ) | 743 ( 60.5 ) | 124 ( 70.9 ) | <0.01^^ | 205 ( 71.7 ) | 106 ( 74.1 ) | 99 ( 69.2 ) | 0.3583^^ |
| **Early use of Methylprednisolone within 24 hours , n (%)** | 152 ( 10.8 ) | 139 ( 11.3 ) | 13 ( 7.4 ) | 0.1221^^ | 27 ( 9.4 ) | 15 ( 10.5 ) | 12 ( 8.4 ) | 0.5441^^ |
| **Early use of Tocilizumab within 24 hours, n (%)** | 301 ( 21.4 ) | 267 ( 21.7 ) | 34 ( 19.4 ) | 0.4886^^ | 47 ( 16.4 ) | 29 ( 20.3 ) | 18 ( 12.6 ) | 0.0792^^ |
| **Proning at admission, n (%)** | 333 ( 24.4 ) | 306 ( 25.6 ) | 27 ( 16.0 ) | <0.01^^ | 56 ( 19.6 ) | 30 ( 21.0 ) | 26 ( 18.2 ) | 0.5511^^ |
| **Mechanical Ventilation within 24 hours of ICU admission, n (%)** | 982 ( 70.3 ) | 852 ( 69.6 ) | 130 ( 75.1 ) | 0.1358^^ | 208 ( 72.7 ) | 101 ( 70.6 ) | 107 ( 74.8 ) | 0.4257^^ |
| **A-A Gradient , Median (Q1,Q3)** | 412.6 (254.07, 560.59) | 416.0 (251.19, 561.04) | 402.2 (290.11, 557.04) | 0.7827^ | 412.0 (281.20, 561.84) | 419.8 (266.81, 572.33) | 409.9 (292.37, 561.75) | 0.9164^ |
| **Oxygenation Index (OI), Median (Q1,Q3)** | 16.1 (9.03, 25.64) | 16.2 (9.46, 27.15) | 14.5 (7.66, 22.76) | 0.1518^ | 16.4 (8.99, 24.51) | 17.4 (12.50, 28.92) | 15.7 (7.69, 22.90) | 0.1266^ |
| **Inotropes/vasopressors use within 24 hours of admission), n(%)** | 344 ( 24.7 ) | 309 ( 25.4 ) | 35 ( 20.3 ) | 0.1532^^ | 58 ( 20.4 ) | 29 ( 20.3 ) | 29 ( 20.4 ) | 0.9761^^ |
| **Vasoactive Inotropic Score, Mean (SD)** | 8.6 (49.08) | 9.4 (52.26) | 3.2 (15.45) | 0.8558^ | 4.2 (29.49) | 4.7 (38.33) | 3.6 (17.00) | 0.5934^ |
| **Lactic acid Baseline, Median (Q1,Q3)** | 1.7 (1.29, 2.43) | 1.7 (1.29, 2.43) | 1.7 (1.30, 2.42) | 0.5879^ | 1.7 (1.24, 2.42) | 1.7 (1.18, 2.51) | 1.7 (1.28, 2.35) | 0.7622^ |
| **Platelets count Baseline, Median (Q1,Q3)** | 242.0 (187.00, 316.00) | 243.0 (188.00, 319.00) | 235.0 (178.00, 303.00) | 0.2446^ | 236.5 (184.00, 311.00) | 237.0 (189.00, 321.00) | 236.0 (173.00, 301.00) | 0.4171^ |
| **Total WBC Baseline, Median (Q1,Q3)** | 9.5 (6.61, 12.90) | 9.5 (6.73, 13.00) | 8.8 (6.19, 12.30) | 0.1726^ | 9.0 (6.30, 12.40) | 9.1 (6.29, 12.50) | 8.8 (6.30, 12.10) | 0.9097^ |
| **International normalized ratio (INR), Median (Q1,Q3)** | 1.1 (1.01, 1.20) | 1.1 (1.01, 1.20) | 1.1 (1.00, 1.15) | 0.0124^ | 1.1 (1.01, 1.15) | 1.1 (1.00, 1.14) | 1.1 (1.02, 1.17) | 0.2136^ |
| **activated partial thromboplastin time (aPTT) Baseline, Median (Q1,Q3)** | 30.1 (26.80, 34.00) | 30.2 (27.00, 34.00) | 29.3 (26.20, 32.70) | 0.0629^ | 29.5 (26.90, 33.05) | 29.9 (27.50, 33.30) | 29.3 (26.40, 32.70) | 0.3081^ |
| **Total bilirubin, Median (Q1,Q3)** | 10.0 (7.10, 15.00) | 10.1 (7.20, 15.20) | 10.0 (7.00, 14.90) | 0.6076^ | 9.9 (7.40, 14.00) | 9.0 (7.20, 12.50) | 11.4 (7.60, 14.90) | 0.2068^ |
| **Alanine transaminase (ALT) at admission, Median (Q1,Q3)** | 37.0 (24.00, 58.00) | 36.0 (23.00, 58.00) | 41.0 (26.00, 58.00) | 0.1000^ | 37.0 (24.00, 57.00) | 35.0 (20.00, 53.00) | 42.0 (26.00, 60.00) | 0.0158^ |
| **Aspartate transaminase (AST) at admission, Median (Q1,Q3)** | 51.5 (34.00, 77.00) | 51.0 (34.00, 77.00) | 53.0 (36.00, 75.00) | 0.4801^ | 51.0 (34.00, 73.00) | 50.0 (32.00, 72.00) | 53.0 (36.50, 80.50) | 0.1818^ |
| **Albumin Baseline, Median (Q1,Q3)** | 33.0 (29.00, 36.00) | 33.0 (29.00, 36.00) | 31.8 (27.50, 34.00) | 0.0004^ | 32.0 (28.00, 35.00) | 32.0 (29.00, 35.00) | 31.0 (27.00, 34.00) | 0.0523^ |
| **Hematocrit at admission, Mean (SD)** | 0.4 (0.34, 0.43) | 0.4 (0.33, 0.43) | 0.4 (0.35, 0.42) | 0.3918^ | 0.4 (0.35, 0.42) | 0.4 (0.34, 0.42) | 0.4 (0.35, 0.42) | 0.2733^ |
| **Creatine phosphokinase (CPK) baseline (U/l), Median (Q1,Q3)** | 168.0 (74.00, 428.00) | 168.0 (74.00, 439.00) | 169.0 (72.00, 405.00) | 0.7407^ | 172.5 (77.00, 508.00) | 174.0 (62.00, 580.00) | 171.0 (81.00, 419.00) | 0.8612^ |
| **C-reactive protein (CRP) baseline (mg/l), Median (Q1,Q3)** | 133.0 (73.00, 203.00) | 135.5 (73.00, 204.12) | 125.0 (78.00, 192.00) | 0.5861^ | 121.0 (72.00, 186.00) | 117.0 (71.00, 176.00) | 126.0 (75.00, 191.50) | 0.5174^ |
| **ESR at admission, Median (Q1,Q3)** | 71.0 (48.00, 94.00) | 70.0 (45.00, 92.00) | 77.0 (51.00, 103.00) | 0.0638^ | 75.0 (55.00, 101.00) | 70.0 (51.00, 96.00) | 77.0 (60.00, 103.00) | 0.2370* |
| **Procalcitonin (ng/ml), Median (Q1,Q3)** | 0.3 (0.14, 1.03) | 0.3 (0.14, 1.08) | 0.3 (0.12, 0.75) | 0.4449^ | 0.3 (0.12, 1.00) | 0.3 (0.11, 1.03) | 0.4 (0.14, 0.95) | 0.7098^ |
| **Fibrinogen Level baseline (gm/l), Median (Q1,Q3)** | 5.5 (3.85, 7.02) | 5.4 (3.77, 7.02) | 5.8 (4.75, 7.32) | 0.0940^ | 5.6 (4.10, 6.99) | 5.0 (3.70, 6.75) | 5.7 (4.54, 7.10) | 0.1869^ |
| **D-dimer Level baseline, Median (Q1,Q3)** | 1.3 (0.73, 3.16) | 1.3 (0.72, 3.19) | 1.3 (0.76, 3.00) | 0.8361^ | 1.3 (0.71, 2.69) | 1.4 (0.72, 2.45) | 1.3 (0.71, 3.02) | 0.8864^ |
| **Ferritin Level baseline, Median (Q1,Q3)** | 687.7 (364.54, 1547.23) | 712.8 (374.30, 1584.00) | 509.7 (345.60, 1112.10) | <0.01^ | 558.5 (366.00, 1273.50) | 747.2 (373.40, 1550.60) | 500.8 (359.90, 1076.60) | 0.1120^ |
| **Blood glucose level Baseline Within 24 hours of ICU admission, Median (Q1,Q3)** | 10.9 (7.60, 15.20) | 11.0 (7.80, 15.20) | 10.1 (7.30, 15.20) | 0.3695^ | 10.2 (7.30, 14.20) | 10.2 (7.50, 14.00) | 10.0 (7.30, 14.70) | 0.8896^ |
| **Best GCS at admission, Median (Q1,Q3)** | 15.0 (14.00, 15.00) | 15.0 (14.00, 15.00) | 15.0 (13.00, 15.00) | 0.6371^ | 15.0 (13.50, 15.00) | 15.0 (14.00, 15.00) | 15.0 (13.00, 15.00) | 0.5287^ |
| **PaO2/FiO2 ratio within 24 hours of admission, Median (Q1,Q3)** | 82.8 (60.86, 135.90) | 83.1 (61.00, 139.25) | 81.6 (58.00, 114.82) | 0.1924^ | 81.1 (58.06, 121.94) | 78.8 (57.60, 122.56) | 81.9 (60.11, 119.83) | 0.7434^ |
| **FIO2 requirement (%) at admission, Median (Q1,Q3)** | 75.0 (50.00, 100.00) | 75.0 (50.00, 100.00) | 70.0 (60.00, 92.50) | 0.6632^ | 70.0 (60.00, 100.00) | 70.0 (50.00, 100.00) | 75.0 (60.00, 100.00) | 0.5202^ |
| **Respiratory rate (Breath Per Minute) at admission, Median (Q1,Q3)** | 28.0 (23.00, 33.00) | 28.0 (23.00, 33.00) | 28.0 (24.00, 33.00) | 0.7986^ | 27.5 (23.00, 33.00) | 28.0 (23.00, 32.00) | 27.0 (23.00, 33.00) | 0.9295^ |
| **Highest heart rate (HR) at admission, Median (Q1,Q3)** | 103.0 (90.00, 115.00) | 103.0 (90.00, 115.00) | 103.0 (91.00, 115.00) | 0.8166^ | 102.0 (90.00, 115.00) | 101.5 (90.00, 116.50) | 102.0 (91.00, 114.00) | 0.9224^ |
| **Lowest MAP at admission, Median (Q1,Q3)** | 72.0 (63.00, 83.00) | 73.0 (63.00, 83.00) | 71.0 (63.00, 80.00) | 0.0602^ | 72.0 (64.00, 82.00) | 73.5 (65.00, 82.00) | 71.0 (63.00, 80.00) | 0.1872^ |
| **Pharmacological DVT prophylaxis use during ICU stay ,n (%)** | 1271 ( 90.9 ) | 1103 ( 90.2 ) | 168 ( 96.0 ) | 0.0123^^ | 272 ( 95.4 ) | 134 ( 94.4 ) | 138 ( 96.5 ) | 0.3872^^ |
| High dose of Pharmacological DVT prophylaxis, n(%)≠ | 488 ( 38.5 ) | 425 ( 38.6 ) | 63 ( 37.7 ) | 0.6372^^ | 125 ( 46.1 ) | 71 ( 53.0 ) | 54 ( 39.4 ) | 0.0786^^ |
| Standard dose of Pharmacological DVT prophylaxis, n(%)≠ | 658 ( 51.9 ) | 567 ( 51.5 ) | 91 ( 54.5 ) | 0.6372^^ | 131 ( 48.3 ) | 57 ( 42.5 ) | 74 ( 54.0 ) | 0.0786^^ |
| Low dose of Pharmacological DVT prophylaxis, n(%)≠ | 121 ( 9.6 ) | 108 ( 9.8 ) | 13 ( 7.8 ) | 0.6372^^ | 15 ( 5.5 ) | 6 ( 4.5 ) | 9 ( 6.6 ) | 0.0786^^ |
| **Aspirin use during ICU stay, n (%)** | 329 ( 23.4 ) | 284 ( 23.1 ) | 45 ( 25.7 ) | 0.4464^^ | 70 ( 24.5 ) | 39 ( 27.3 ) | 31 ( 21.7 ) | 0.2712^^ |
| **Patient received nephrotoxic drugs/material during ICU stay, n (%)***$ | 1187 ( 85.0 ) | 1022 ( 83.7 ) | 165 ( 94.3 ) | 0.0002^^ | 256 ( 89.5 ) | 122 ( 85.3 ) | 134 ( 93.7 ) | 0.0206^^ |
| *T Test / ^ Wilcoxon rank sum test is used to calculate the P-value.  ^^ Chi square/ ** Fisher’s Exact teat is used to calculate P-value.  *$ Nephrotoxic medications/ material included IV Vancomycin, Gentamicin, Amikacin, Contrast, Colistin, Furosemide, and/or Sulfamethoxazole/trimethoprim  ≠ Patients who received either Enoxaparin 40 mg daily or UFH 5000 Unit three times daily were grouped under the "standard dose VTE prophylaxis. Any patient who received higher than standard dose but not as treatment dose (Enoxaparin 1mg/kg q12hr or 1.5mg/kg q24hr or UFH infusion) was categorized as receiving "High VTE prophylaxis dose". On the other hand, lower VTE prophylaxis considered for patient who received Enoxaparin <40 mg/day or Unfractionated heparin (UFH ) <5000 Units three times daily/day). | | | | | | | | |
